# Country of origin disparities in sub-optimal menstrual hygiene management and intersecting reproductive health concerns: a pilot study from the Dominican republic

**DOI:** 10.3389/frph.2025.1702366

**Published:** 2025-12-15

**Authors:** Madison Douglas, Kelly Dressel, Zoe Kusinitz, Supriya D. Mehta, Stephanie Crane

**Affiliations:** Rush University Medical Center, Office of Global Health, Rush University, Chicago, IL, United States

**Keywords:** menstrual health, menstrual hygiene management, women's health, global health, migrant health, health equity

## Abstract

**Introduction:**

Due to long-standing systemic xenophobia in the Dominican Republic (DR), women of Haitian descent are underrepresented in national health data. This study examined menstrual hygiene management (MHM) and other reproductive health concerns in a migrant-dense community to inform future health studies and potential interventions.

**Methods:**

An anonymous cross-sectional survey was offered in a low-income community near Santo Domingo over two, one-week periods from October 2023 to April 2024. Eligible participants were at least 14 years old. Reproductive health-related factors were assessed among participants who had menstruated in the last 12 months. Multivariable adjusted modified Poisson regression was used to identify factors associated with sub-optimal MHM, defined by lack of water and/or soap, privacy, or safe menstrual products.

**Results:**

Among 148 participants who menstruated in the past 12 months, over half (53.0%) reported that menstrual materials were unaffordable sometimes (43.5%) or always (9.5%), and more than one-third (38.1%) reported menses interfering with regular duties. Overall, 21.6% had sub-optimal MHM; although 94% of women reported using disposable pads to manage menses, 14.2% were also using cloth (*n* = 19) or underwear/diapers (*n* = 2). Other factors contributing to sub-optimal MHM were lack of privacy (4.1%) and lack of soap (8.7%). In analyses adjusted for age, educational attainment, employment status, and menstrual product affordability, Haitian-born women were more likely to have sub-optimal MHM (aPR = 7.25; 95% CI 4.23–12.4). Compared to women with optimal MHM, women with sub-optimal MHM were more likely to report “Poor” general health (46.9% vs. 19.8%, *p* = 0.004) and menses interfering with regular duties (56.3% vs. 32.8%, *p* = 0.015), and were less likely to report reliable contraceptive use (43.3% vs. 68.5%, *p* = 0.011) and prenatal care at last pregnancy (79.3% vs. 95.3%, *p* = 0.005). Haitian-born women were also more likely to have intersecting sub-optimal MHM and contraceptive gap or lack of prior prenatal care (20.4% vs. 2.2%, *p* = 0.001).

**Conclusions:**

Sub-optimal MHM and its association with other reproductive health concerns was common in this migrant-dense community, notably appearing more frequently among Haitian-born women. Our preliminary findings suggest opportunities for future research and context-appropriate approaches addressing structural barriers to menstrual and reproductive health—particularly for migrant populations

## 1 Introduction

Period poverty—defined as limited access to affordable menstrual products, adequate hygiene facilities, and menstrual health education—is a prevalent public health issue in the Dominican Republic (DR) that disproportionately affects low-income women and girls. A recent survey in rural DR found that 39.7% of women and girls cannot afford to buy menstrual products (1). Menstrual hygiene products are not subsidized or treated as essential goods, which limits their affordability and disproportionately affects individuals with fewer financial resources. Some resort to unsafe alternatives such as cloth rags or toilet paper, which can cause infections, chronic discomfort, and other health complications (2, 3). The same survey revealed that 45.2% of girls in rural DR do not know why menstruation occurs (1). Menstrual education is often neglected or underemphasized in schools, which perpetuates myths, taboos, and the association of menses with shame or impurity (4, 5). Sub-optimal access to menstrual health resources and cultural stigmas subsequently forces 20% of girls in rural DR to miss 2–3 days of school every month (6), hindering their education and future opportunities. In the DR, data on period poverty and menstrual health disparities is limited and has not been evaluated in conjunction with other reproductive health concerns.

While the prevalence of contraceptive use in the DR is among the highest in Latin America and comparable to that of the United States (7, 8), teenage pregnancy rates are on the rise (9). The DR has the highest rate of early marriage and adolescent pregnancy in the Caribbean, which poses significant health risks and restricts future opportunities and access to basic rights for women (10). An estimated two-thirds of students in the DR are not receiving comprehensive sexuality education, leaving adolescents without essential information about safe sexual practices, healthy relationships, and their human rights (11). The Ministry of Education has been slow to implement a new approach to comprehensive sexuality education, believed to be due to opposition from the Catholic Church hierarchy and other socially conservative groups (12). Adolescent mothers may find it difficult or impossible to continue their education and instead engage in unpaid household activities (i.e., caregiving, cooking, cleaning), perpetuating a generational cycle of poverty among women. Current barriers to contraceptive use include limited access to family planning services, misinformation, cultural myths and taboos, and male partner influence on reproductive decision-making.

There is little research on the intersection of period poverty and contraceptive use in the DR's low-income, migrant-dense communities. Hundreds of Dominican “bateyes,” or shanty-town camps, were originally constructed in the early 1900s for Haitian sugarcane cutters who had migrated across the border to find work. Over the decades, many of these workers did not return to Haiti at season end, creating a large, permanent, and unwelcomed population of Haitians in the DR. Currently, most immigrants in the DR are from Haiti (13). In recent years, hundreds of thousands of Haitians have migrated to the DR to escape poverty, rampant violence, and political unrest. This troubled history of political and migratory conflict has fueled anti-immigrant sentiment, institutional racism, and xenophobia in the DR for the last century (14). Most bateyes in the DR have not changed significantly since their erection; many are still without running water, electricity, cooking facilities, bathrooms, schools, or medical facilities. The Dominican Constitution does not extend citizenship to Haitian migrants nor children born to non-naturalized Haitian parents, which has given rise to the most widespread statelessness in the Americas (15). This community faces racial profiling, structural discrimination, and mass deportation without due process of law, and families are confined to generational cycles in this permanent underclass. They have no legal status in the DR with no right to own property, no right to an education, no right to vote, and no right to healthcare. Thus, women in these migrant-dense communities endure substandard access to reproductive health services such as family planning and maternal and prenatal care (14) and remain continually underrepresented in national women's health data. Our project aimed to evaluate menstrual hygiene management, contraceptive use, and other reproductive health challenges in an underserved, migrant-dense community to identify factors associated with sub-optimal practices in these domains. The findings from this study will guide the future development and strategic allocation of reproductive health interventions tailored to the needs of this community.

## 2 Methods

### 2.1 Recruitment and eligibility

Participants were recruited from the community by voluntary self-referral during two, one-week periods (October 15–21, 2023, and April 14–20, 2024). Inclusion criteria included emancipated women 14–17 years old (i.e., have children, work full time, and/or married/living as married) or women at least 18 years old, who had menstruated in the past 12 months, or who had not menstruated in the past 12 months due to current breastfeeding, pregnancy, or hormonal contraceptive use. Exclusion criteria included men, women less than 14 years old, unemancipated women less than 18 years old, and women who had not menstruated in the past 12 months due to menopause. All participants provided verbal informed consent in their preferred language (Spanish or Creole) before enrollment in the study.

### 2.2 Data collection

Participants completed the survey questionnaire orally in their preferred language (Spanish or Creole) with a medical translator and surveyor from the research team. The questionnaire collected data about demographics, menstruation management, reproductive history, contraceptive and sexual practices, and fertility preferences. A 12-month timeframe was used to determine menstruation status and to identify women whose lack of menses was due to breastfeeding, pregnancy, or hormonal contraception; all other contraceptive variables and menstruation practices utilized a 6-month recall period. Study data were collected and managed using REDCap (Research Electronic Data Capture) electronic data capture tools hosted at Rush University Medical Center (16, 17).

The survey was conducted in two rounds in distinct neighboring communities. In the first round, research staff were stationed outside a local primary care clinic where women were already presenting for routine visits. Staff briefly introduced the study to patients and invited those who were eligible and interested to participate. In the second round, recruitment occurred directly within the community. Local staff and community leaders helped disseminate information about the study through word-of-mouth and household outreach. Small groups of women were invited to a nearby meeting point, where research staff met them in person. Surveys were then completed individually in a private area with a member of the research team. Because no identifying information was collected, we could not formally screen for duplicate participation. However, research staff familiar with both communities supervised recruitment and data collection, making repeat participation unlikely.

### 2.3 Analysis

The primary outcome for analysis was sub-optimal menstrual hygiene management (MHM). Poor materials for managing menses included use of cloth, tissue paper, toilet paper, paper towels, cotton balls, underwear only, or diaper. These materials were not mutually exclusive; if a participant reported using both safe and poor materials, they were classified as using poor materials. To assess privacy, women were asked if at their last period they were able to change menstrual management materials in privacy. Lastly, women were asked if during their last period they had access to soap, water, or both whenever needed. Sub-optimal MHM was defined as any of the following: poor materials for managing menses, lack of privacy, or lack of soap or water. Supplementary Figure S1 summarizes the overlap of these.

We compared the distribution of covariates that varied by sub-optimal vs. sufficient MHM via chi-square tests (Fisher's exact test was applied when cell sizes were *n* < 5). Variables *a priori* hypothesized to be associated with sub-optimal MHM (country of origin, age, education, employment status; Supplementary Figure S2), and those with a *p*-value <0.20 were entered in multivariable Poisson regression with robust variance estimation to calculate prevalence ratios and 95% confidence intervals (95% CI). Prior to model building, we examined the correlations among explanatory variables, which ranged from 0.05 to 0.42 by either Spearman or tetrachoric rho, demonstrating no concern for multicollinearity. Statistical analyses were conducted using Stata/SE v18.0 (Stata Corp., College Station, Texas).

To assess the intersection of sub-optimal MHM with other health and reproductive health concerns, we examined the distributions of self-rated general health, reliable contraceptive use, prenatal care, contraceptive gap, and decision making around contraception in relation to sub-optimal MHM. Reliable contraception was defined as using injectable, implant, birth control pill, sterilization, or intrauterine device (IUD). Contraceptive gap was defined as women who were not pregnant, not seeking to become pregnant, and were not using a reliable contraceptive method. We examined these intersecting conditions descriptively, as they would not be causal to sub-optimal MHM, to explore the constellation of sub-optimal reproductive health conditions.

## 3 Results

168 women in the targeted geographic area consented to and completed the survey. 148 had menstruated in the past 12 months, of which 61.5% were born in the DR and most of the remaining were born in Haiti (37.2%). Participants were mean age 30.6 years. Respondents had been living in the community for an average of 13.9 years, with the majority having moved there for family reunification or employment reasons. Most self-reported their general health as “average,” but 25.7% stated that their general health was “poor.” While most respondents had attended secondary school or higher education, 29.1% had only attended primary school or no school at all. Less than half (41.2%) were formally employed.

### 3.1 Menstrual hygiene management

Most (92.2%) respondents had access to clean water, soap, and privacy to change their menstrual products (Table 1). Most used disposable sanitary pads to absorb their menstrual blood (93.9%), but 12% also used cloth. Because sub-optimal MHM was a composite measure and menstrual materials were not mutually exclusive, women could be classified as having sub-optimal MHM if they used disposable pads in conjunction with an unsafe material, if they lacked privacy, or had inadequate access to soap and/or water (Supplementary Figure S1 displays the overlap of these). Money to purchase menstrual supplies was most often provided by the respondent or their partner. Over half (52%) of respondents reported that menstrual supplies were “sometimes” or “always” unaffordable, and more than a third (37.8%) reported their menses interfered with their regular duties.

**Table 1:** Distribution of participant characteristics overall and by menstrual hygiene management (MHM) status.

Variable	Total, *N* = 148	Sufficient MHM, *N* = 116	Sub-Optimal MHM, *N* = 32	*P*-Value
	*n* (%)	*n* (%)	*n* (%)	
Place of Birth				<0.001
Dominican Republic^a^	93 (62.8)	88 (75.9)	5 (15.6)	
Haiti	55 (37.2)	28 (24.1)	27 (84.4)	
Median years living in community (IQR) (*Missing* *n* = 4)	11.5 (4–21)	14 (6–23.8)	4 (1.25–11.8)	<0.001
Median Age in Years (IQR) (*Missing* *n* = 4)	29.0 (23.2–36)	30 (23–35.5)	29 (25–40)	0.449
Educational Attainment				0.039
Some secondary or more	105 (71.0)	87 (75.0)	18 (56.2)	
Primary or less	43 (29.0)	29 (25.0)	14 (43.8)	
Employment status (*Missing n* = 1)				0.083
Not employed	86 (58.5)	63 (54.8)	23 (71.9)	
Employed	61 (41.5)	52 (45.2)	9 (28.1)	
Person providing money to purchase menstrual materials				0.002
Self (includes *n* = 1, mother also pays)	71 (49.6)	56 (60.2)	15 (30.0)	
Husband/boyfriend/partner	46 (32.2)	21 (22.6)	25 (50.0)	
Self and husband/boyfriend/partner	15 (10.5)	8 (8.6)	7 (14.0)	
Other (mother, relative, friend)	11 (7.7)	8 (8.6)	3 (6.0)	
Frequency that menstrual materials are unaffordable				0.017
Never unaffordable	70 (47.3)	51 (54.8)	19 (34.6)	
Sometimes or always unaffordable	78 (52.7)	42 (45.2)	36 (65.4)	
Menstrual period interfered with regular duties				0.069
No	92 (62.2)	63 (67.7)	29 (52.7)	
Yes	56 (37.8)	30 (32.3)	26 (47.3)	
Variables used to define sub-optimal MHM^b^				
Materials used to manage menses^c^				
Reusable pads	1 (0.7)	1 (0.9)	0 (0)	
Disposable pads	139 (93.9)	114 (98.3)	25 (78.1)	
Tampons	1 (0.7)	1 (0.9)	0 (0)	
Cloth	19 (12.8)	0 (0)	19 (59.4)	
Underwear only, Diaper	2 (1.4)	0 (0)	2 (6.2)	
Privacy at last period				
Yes	142 (96.0)	114 (100)	26 (81.3)	
No	6 (4.0)	0 (0)	6 (18.7)	
Soap and water available				
Soap and water	135 (91.2)	116 (100)	19 (59.4)	
Water only	13 (8.8)	0 (0)	13 (40.6)	

a Includes one participant from Bolivia.

b *p*-values not calculated because the variable is included in the definition of MHM.

c Not mutually exclusive.

### 3.2 Other reproductive health concerns

Nearly two-thirds of participants (63.0%) reported use of reliable contraceptives; the most common included injectables, female sterilization (tubal ligation), and the pill which were typically administered by government hospitals or clinics and local pharmacies. For respondents using multiple methods, injectables were the preferred method. Typically, these were administered by a health care provider or a resident in the community. Among the 148 participants who were included in analyses, 10 were currently pregnant. Among those who were not pregnant, 16 (11.6%) reported they were currently trying or wanting to become pregnant. Among 122 participants who were not pregnant and not trying or wanting to become pregnant, 37 (29.8%) had a contraceptive gap, meaning they were not using a reliable contraceptive.

### 3.3 Factors associated with Sub-optimal MHM

In crude analyses, women who were Haitian born, with primary education or less, unemployed, or reported menstrual products to be unaffordable were more likely to have sub-optimal MHM. In analyses adjusted for age, educational attainment, employment status, and menstrual product affordability, Haitian born women remained more likely to have sub-optimal MHM (aPR = 7.25; 95% CI 4.23–12.4) (Table 2).

**Table 2:** Results of crude and multivariable regression: factors associated with sub-optimal menstrual hygiene management (MHM).

Variable	Crude prevalence ratio (95% CI), *p*-value	Adjusted, *N* = 144^a^ prevalence ratio (95% CI), *p*-value
Haitian Born (vs. Dominican Republic)	9.13 (6.46–12.9),<0.001	7.25 (4.23–12.4),<0.001
Median Age in Years	1.01 (0.96–1.07), 0.574	1.01 (0.98–1.05), 0.447
Primary or less educational attainment (vs. some secondary or more)	1.90 (1.042.2064–3.47), <0.001	1.13 (0.66–1.94), 0.662
Unemployed (vs. employed)	1.81 (0.67–4.91), 0.242	1.25 (0.98–2.69), 0.077
Past 6 months: Menstrual products unaffordable sometimes or always (vs. never)	2.69 (1.15–6.28), 0.022	1.81 (1.22–2.69), 0.003

a Age is missing for *n* = 4.

### 3.4 Intersection of Sub-optimal MHM with other reproductive health concerns

Table 3 shows that compared to women with sufficient MHM, women with sub-optimal MHM were more likely to report their health status as poor (46.9% vs. 19.8%, *p* = 0.004), less likely to report using reliable contraception (43.3% vs. 68.5%, *p* = 0.011), less likely to report prenatal care at last pregnancy (79.3% vs. 95.3%, *p* = 0.005), and more likely to report contraceptive gap (50% vs. 25%, *p* = 0.014).

**Table 3:** Distribution of health and reproductive health factors by sub-optimal menstrual hygiene management (MHM) and country of birth.

Variable	Sufficient MHM, *N* = 116 *n* (%)	Sub-optimal MHM, *N* = 32 *n* (%)	*p*-value	Dominican, *N* = 93 *n* (%)	Haitian, *N* = 55 n (%)	*p*-value
Health status						
Poor	23 (19.8)	15 (46.9)	0.004	10 (10.1)	28 (50.9)	<0.001
Average	80 (69.0)	17 (53.2)		71 (76.3)	26 (47.3)	
Excellent	13 (11.2)	0 (0)		12 (12.9)	1 (1.8)	
Reliable contraception: Injectable, implant, sterilization, IUD, or pill						
No	34 (31.5)	17 (56.7)	0.011	29 (33.0)	22 (44.0)	0.196
Yes	74 (68.5)	13 (43.3)		59 (67.0)	28 (56.0)	
Prenatal care at last pregnancy^a^						
No	5 (4.7)	6 (20.7)	0.005	3 (3.6)	8 (15.4)	0.021
Yes	102 (95.3)	23 (79.3)		81 (96.4)	44 (84.6)	
Contraceptive gap: Not pregnant, does not want to get pregnant, not using reliable contraceptive						
No (no contraceptive gap)	72 (75.0)	13 (50.0)	0.014	57 (74.0)	28 (62.2)	0.171
Yes (contraceptive gap)	24 (25.0)	13 (50.0)		20 (26.0)	17 (37.8)	
Who usually makes the decision on whether or not you should use contraception						
Self	72 (62.1)	16 (50.0)	0.148	62 (66.6)	26 (47.3)	0.004
Husband/boyfriend	8 (6.9)	3 (9.4)		2 (2.2)	9 (16.4)	
Jointly with husband/boyfriend	34 (29.3)	10 (31.2)		27 (29.0)	17 (30.9)	
Other	2 (1.7)	3 (9.4)		2 (2.2)	3 (5.4)	

a Excludes participants who report being not currently pregnant and never having been pregnant.

**Figure 1:**
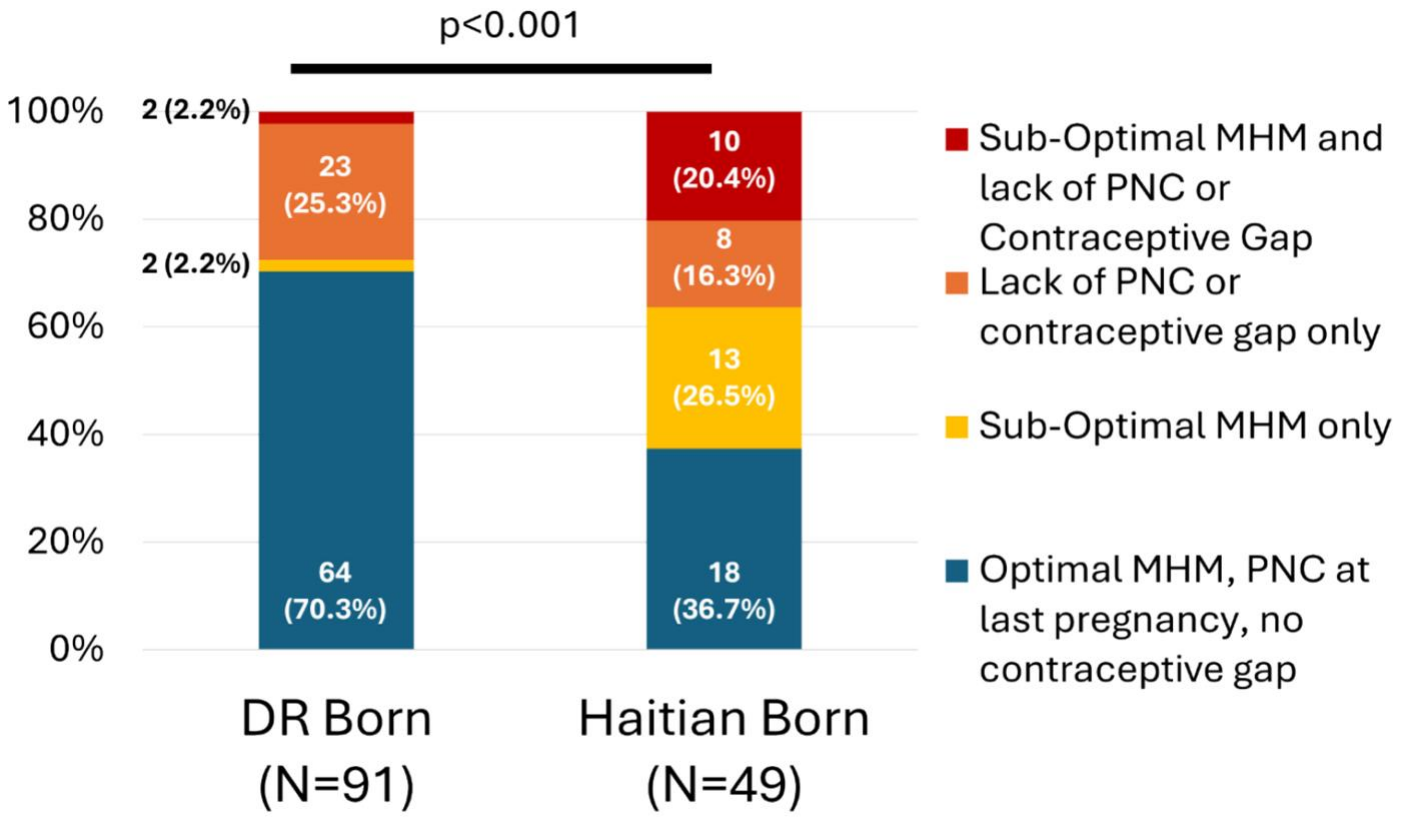
The proportion of participants with combinations of sub-optimal menstrual hygiene management, lack of prenatal care (PNC) at last pregnancy (if applicable), and/or with contraceptive gap are shown stratified by participants, country of birth.

Figure 1 shows the overlap between sub-optimal MHM, lack of prenatal care at last pregnancy, and contraceptive gaps. Compared with women born in the DR, Haitian-born participants were far less likely to meet all three optimal conditions (36.7% vs. 70.3%). They were also more likely to experience sub-optimal MHM alone (26.5% vs. 2.2%) or to have sub-optimal MHM that intersected with lack of prenatal care or a contraceptive gap (20.4% vs. 2.2%). Overall differences between groups were statistically significant (*p* < 0.001, Fisher's exact test).

## 4 Discussion

In our small pilot survey of menstruating women living in a low-income community in the DR, 21.6% reported sub-optimal MHM. This often intersected with broader reproductive health concerns including lack of prenatal care at last pregnancy, use of reliable contraceptives, and contraceptive gap. These findings of co-occurrence highlight that women with sub-optimal MHM, an important public health concern on its own, may face associated reproductive health needs. Consistent with studies from other low-income countries (18–21), we found that menstruating women in this community experienced high rates of disruption to work and school. This is a global public health issue linked to insufficient menstruation education and stigma, unaffordability of menstrual hygiene products, and lack of adequate hygiene facilities (22). Low-income individuals are particularly vulnerable to period poverty due to the financial burden of purchasing menstrual supplies and underwear used during menstruation, leading many to rely on unsafe alternatives due to cost constraints as observed in our study. This mirrors global reports from the World Health Organization (WHO) and United Nations Children's Fund (UNICEF) emphasizing that inadequate access to menstruation materials and sanitation facilities disproportionately affects women in low-income communities, contributing to health risks, social stigma, and disruptions to education and work (23).

Our finding show that Haitian-born respondents reported higher rates of sub-optimal MHM compared to Dominican-born respondents. Importantly, this disparity must be understood within the broader structural context of anti-Haitian racism and xenophobia in the DR. Haitian migrants and their descendants face long-standing systemic exclusion from citizenship, formal employment and education, and public insurance coverage, which collectively shape access to water, sanitation, healthcare, and other basic services. These structural barriers likely contribute to the markedly higher prevalence of sub-optimal MHM observed among Haitian-born women in this pilot study (aPR = 7.25) and reflects the cumulative impact of policy-level inequities rather than individual factors alone. While our data cannot infer causality, the direction and magnitude of the association align with well-documented patterns of structural marginalization. Previous studies have observed rates of poor menstrual hygiene globally among migrants and refugees. For example, in refugee camps and other humanitarian settings, menstruators face significant challenges due to inadequate water, sanitation, and hygiene (WASH) infrastructure (24–26). Additionally, lack of privacy, inadequate lighting, and poor accessibility have been linked to difficulties managing menstrual health with dignity and feelings of insecurity (27).

Educational attainment did not remain statistically significant in adjusted analyses, likely because it was correlated with birth country. Haitian-born women were disproportionately represented among those with lower education levels (43.3% vs. 23.8%), making it difficult to disentangle the independent contribution of education once birth country was included in the model. This pattern reflects broader structural inequities, as Haitian migrants and their descendants have historically faced restricted access to schooling, documentation, and public resources in the DR, thus shaping educational trajectories long before adulthood. Nevertheless, educational attainment is an important consideration when designing MHM programs. Previous reports show that lower education levels correlate with a lack of knowledge about menstruation and limited access to menstrual products (28, 29). Women with less education may also have fewer opportunities to advocate for their health needs–an inequity that may be compounded for Haitian-born women who already face social exclusion, language barriers, and discrimination in institutional settings. A recent study conducted in a similar Dominican community showed that parents were the most trusted source of menstrual health information among adolescents (30); however, mothers' own knowledge may be incomplete (31, 32), possibly contributing to ongoing cultural myths and misinformation (33). Our survey did not assess menstrual knowledge or sources of education, stigmas and myths; future research should explore these domains to inform culturally relevant messaging. Long-term efforts should also include assessing WASH infrastructure, evaluating menstrual knowledge, and improving access to affordable and durable menstrual products such as menstrual cups.

Given the intersectionality we observed between sub-optimal MHM and other reproductive health indicators including contraceptive gaps, it is important to interpret MHM within the broader reproductive health landscape of this community. Just under two-thirds (65.5%) of women in our study reported using reliable contraception, slightly less than the overall national rate of 71.8% (7) and slightly above the rate of 61% specifically among low-income, uninsured women in the DR (34). More than a quarter (26.3%) of non-pregnant respondents in this community had an unmet need for contraception, meaning they wished to delay or prevent pregnancy but were not contracepting with a reliable method (e.g., injectable, implant, sterilization procedure, intrauterine device, or birth control pill). This gap is wider than national estimates (35) and may be linked to low income, rural residence, and low education—factors associated with lower rates of modern contraceptive use (36, 37). Young women and girls in the DR are also frequently shamed, stigmatized, or denied access if not accompanied by an adult when seeking sexual and reproductive health services (12), which may contribute to lower use among adolescents (38). These patterns reflect broader structural and socioeconomic barriers including poverty, limited insurance coverage, and social stigma that are known to shape reproductive health access.

As discussed, Haitian migrants and their descendants have no pathway to Dominican citizenship, disqualifying them from public insurance coverage. Reliable contraception methods (e.g., female sterilization, intrauterine devices, implants, and oral and injectable hormones) are typically covered by insurance plans in the DR, but even the publicly-insured face challenges with contraceptive access due to persistent stockouts of certain methods, increasing out-of-pocket costs and further widening the gap of unmet need (34). This leaves uninsured women in the lowest income groups particularly vulnerable to inadequate contraception access (34). Thus, we expected to observe a disparity in contraception access among Haitian-born and Dominican-born participants. However, in our study, although rates of reliable contraception use and unmet contraception need were lower for Haitian born women, the difference was not statistically significant, and could be due to small sample size.

Hormone injectables were the most common method of reliable contraception in this community, followed by female sterilization and the pill. Female sterilization, or tubal ligation, is the most used method in the DR nationally (39). Injectables may be more popular in this community due to their relatively low cost, infrequent dosing, and ease of use compared to daily methods. However, it is important to note that these reversible contraceptive methods can fail—approximately 45% of unintended pregnancies in low- and middle-income countries are attributed to contraceptive failure (40). This includes method-related failures, where the contraceptive itself does not work as expected, and user-related failures, such as incorrect or inconsistent use. Given the wide use of injectables and informal administration practices reported in the community, it is plausible that contraceptive failure may be more common than expected. These informal administration practices may reflect underlying structural barriers to consistent access to trained personnel, insurance coverage, and clinic-based services, which can contribute to user-related failures. However, this remains speculative and warrants further study. The community may also benefit from wider availability of long-acting reversible contraceptives (e.g., IUDs and implants), as these are more reliable and require less maintenance long-term.

A notable portion of unsterilized women who did not desire fertility were unlikely to contracept in the future due to unwanted side effects, religious prohibition, partner opposition, infrequent or no sex, or other reasons. Side effects and health concerns are the most frequently cited reasons for women opting out of hormonal contraception (41). Thus, it is critical to improve reproductive education and counseling for women and couples so they can make informed choices that consider individual circumstances, preferences, and associated risks and benefits with different methods (39). However, several respondents reported a lack of autonomy or joint decision-making when faced with these choices, and several had been coerced to become pregnant. While recent research demonstrates a potential shift toward equal power distribution between partners in the DR (42), it is a traditionally patriarchal culture that favors male dominance, traditional gender roles, and female subordination perpetuated by Catholic values (43, 44). Efforts to address gender norms, relationship power imbalances, and structural inequities by engaging men and promoting joint reproductive decision-making may help address these barriers.

Our study is one of few to report on MHM and reproductive health practices in a low-income, migrant-dense community in the DR, and it is one of the first to explore differences between Dominican-born and Haitian-born women. As a small, cross-sectional pilot study, our findings should be interpreted as preliminary and hypothesis-generating rather than definitive. Given the overlap we observed between sub-optimal MHM and other reproductive health indicators, including contraceptive gaps and limited prenatal care, it is important to situate MHM within the broader reproductive health landscape of this community. Strengthening menstrual health resources through affordable and more durable menstrual products, improving WASH infrastructure, and evaluating menstrual knowledge and stigma to inform culturally relevant education may represent promising avenues for future intervention, pending further study. Additionally, addressing inconsistent access to reliable contraception, failure risks related to informal administration, and gender-based constraints on reproductive autonomy may also be important considerations for reducing compounded vulnerabilities in this community—particularly among migrant women. However, these areas require additional research to determine effective and context-appropriate approaches.

### 4.1 Limitations

Our survey excluded unemancipated adolescents under 18, a group with distinct reproductive health needs and risks in the DR. Given the importance of menstrual hygiene and contraception for this population, their inclusion in future study should be considered and balanced against potential loss of confidentiality requiring guardian assent. Additionally, selection bias could have influenced our findings since participants were recruited through word-of-mouth and voluntary participation. This may have led to a non-representative sample, capturing only certain social groups or individuals with stronger interests in reproductive health. The anonymous design also prevented verification of duplicate participation across survey rounds. Although field staff reported no clear indications of repeat participants, this possibility cannot be excluded and may introduce a small degree of error. Additionally, our study provides insight for local interventions, but its small, convenience-based sample limits generalizability. Larger, more representative studies are needed to confirm these preliminary findings.

### 4.2 Conclusions

In this low-income, migrant-dense community in the DR, Haitian-born women were more likely to reportmenstrual health inequities, including limited access to affordable menstrual materials, disruptions to daily responsibilities due to menses, and sub-optimal MHM. Importantly, sub-optimal MHM was associated with broader reproductive health challenges and may place additional strain on this population. The preliminary findings from this small, cross-sectional pilot study highlights potential areas for further inquiry and reinforce the need for improved access to menstrual hygiene resources and further examination of how structural barriers—including limited WASH infrastructure, legal exclusion, and inequitable access to healthcare and contraception—may shape reproductive health experiences in this community. Future studies with larger, more representative samples are necessary to confirm these associations and guide the development of integrated, context-appropriate interventions—particularly for migrant women.

## Data Availability

The original contributions presented in the study are included in the article/Supplementary Material, further inquiries can be directed to the corresponding author. The datasets are also publicly available at https://doi.org/10.6084/m9.figshare.30670880.

## Supplementary material

Supplementary Figure 1 Proportional Venn diagram showing the intersection of the three criteria contributing to sub-optimal menstrual hygiene management (MHM). Of 32 respondents, 21 reported poor materials, 13 reported no soap, and 6 reported no privacy at their last period. Respondents overlapped with poor materials and no soap (n = 4), poor materials and no privacy (n = 4), no soap and no privacy (n = 2), or all three conditions (n = 2).

Supplementary Figure 2 Conceptual model for factors associated with sub-optimal menstrual hygiene management (MHM). We hypothesized that women born in Haiti would be less likely to have higher educational attainment and employment, and that these would also vary by age. This is based on previous meta-analyses showing that higher educational attainment and economic status are associated with increased likelihood of adequate MHM [1]. We also hypothesized that the relationship between employment status and use of sub-optimal MHM would be mediated by menstrual product affordability - specifically that unemployed women would be more likely to report unaffordability of menstrual products, leading to increased likelihood of sub-optimal MHM. 1. Anbesu EW, Asgedom DK. Menstrual hygiene practice and associated factors among adolescent girls in sub-Saharan Africa: a systematic review and meta-analysis. BMC Public Health. 2023;23(1):33.
