# Supplementary figures and images for: Country of origin disparities in sub-optimal menstrual hygiene management and intersecting reproductive health concerns: a pilot study from the Dominican republic
Source: Front Reprod Health. 2025 Dec 15;7:1702366. doi: 10.3389/frph.2025.1702366 (PMC12745460; doi:10.3389/frph.2025.1702366)

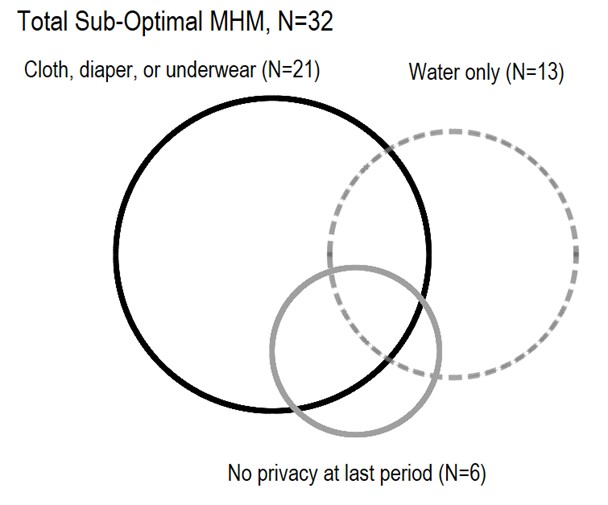

Supplement: Supplementary Figure 1 — Proportional Venn diagram showing the intersection of the three criteria contributing to sub-optimal menstrual hygiene management (MHM). Of 32 respondents, 21 reported poor materials, 13 reported no soap, and 6 reported no privacy at their last period. Respondents overlapped with poor materials and no soap (n = 4), poor materials and no privacy (n = 4), no soap and no privacy (n = 2), or all three conditions (n = 2). [file Image1.jpg]

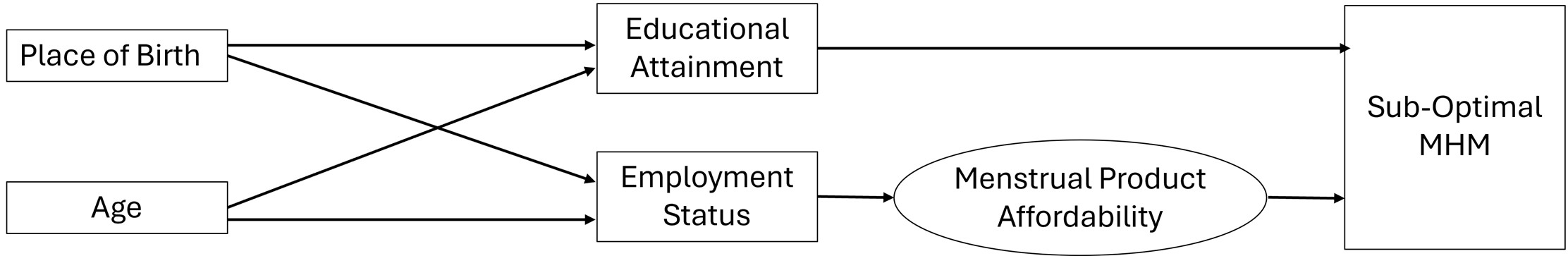

Supplement: Supplementary Figure 2 — Conceptual model for factors associated with sub-optimal menstrual hygiene management (MHM). We hypothesized that women born in Haiti would be less likely to have higher educational attainment and employment, and that these would also vary by age. This is based on previous meta-analyses showing that higher educational attainment and economic status are associated with increased likelihood of adequate MHM [1]. We also hypothesized that the relationship between employment status and use of sub-optimal MHM would be mediated by menstrual product affordability - specifically that unemployed women would be more likely to report unaffordability of menstrual products, leading to increased likelihood of sub-optimal MHM. 1. Anbesu EW, Asgedom DK. Menstrual hygiene practice and associated factors among adolescent girls in sub-Saharan Africa: a systematic review and meta-analysis. BMC Public Health. 2023;23(1):33. [file Image2.jpg]
